# Negative affect interacts with perceptual affective biases

**DOI:** 10.1177/02698811251409143

**Published:** 2026-02-01

**Authors:** Thomas Murray, Rebecca P. Lawson

**Affiliations:** 1 Department of Psychology, University of Cambridge, UK

**Keywords:** affective biases, mood disorders, negative affect, visual adaptation, metacognition

## Abstract

**Background:**

Affective biases are central to mood and anxiety disorders, with individuals often interpreting ambiguous facial expressions more negatively. Adaptation paradigms, where exposure to emotional stimuli shifts perception, provide a tool to separate perceptual from decisional biases, but have not been used to study emotional biases relevant to affective disorders.

**Aims:**

To determine whether affective biases in facial emotion perception arise from perceptual or decisional processes, and to examine how these biases are modulated by individual differences in negative affect.

**Methods:**

Eighty participants completed emotion and identity discrimination tasks before and after adaptation. Participants made binary judgements of morphed facial expressions (happy/sad) and identities (Bonny/Sheila), followed by confidence ratings. Logistic and Gaussian functions were used to estimate adaptation effects from shifts in the point of subjective equality (PSE) and peak uncertainty.

**Results/Outcomes:**

Adaptation produced repulsive aftereffects: exposure to happy faces biased perception towards sadness, and vice versa, with analogous effects for identity. Correlated shifts in PSE and uncertainty indicated a perceptual rather than decisional origin. Negative affect (derived from Beck Depression Inventory and State Trait Anxiety Inventory-trait questionnaires) moderated this relationship, such that individuals higher in negative affect showed stronger perceptual biases towards sadness.

**Conclusions/Interpretation:**

Findings suggest that negative affect modulates low-level perceptual encoding of emotional expressions. This supports cognitive neuropsychological models positing that antidepressants first target early perceptual biases and highlight perceptual encoding as a potential mechanism underlying affective biases in mood and anxiety disorders.

## Introduction

Gauging people’s emotional state from their facial expressions is vital for social interactions. People must make inferences of unobservable emotional states using observable facial expressions of emotion (Murray et al., 2024; Ong et al., 2019). As the emotional state of the individual is hidden, these inferences can be subject to biases, which can be caused by contextual factors (Carrera-Levillain and Fernandez-Dols, 1994; Carroll and Russell, 1996; Tanaka-Matsumi et al., 1995), hormonal states (Di Simplicio and Harmer, 2016; Pearson and Lewis, 2005) and the mood of the observer (Dyer et al., 2022). Importantly, such biases can occur frequently in neuropsychiatric conditions, such as depression and anxiety, where they are thought to contribute to altered emotional processing and impaired social functioning (Saris et al., 2017).

Affective biases play a key role in mood and anxiety disorders, and are thought to contribute to the development and maintenance of these conditions (Harmer et al., 2009). Cognitive neuropsychological models describe how symptoms of depression and anxiety arise as states learned from dysfunctional negative schemata, which have been formed and maintained by low- and high-level affective processing biases (Bishop, 2007; Harmer et al., 2009; Roiser et al., 2012). Low-level biases occur in early, automatic stages of processing, such as the perceptual encoding of emotional stimuli, and are thought to result from altered processing in visual processing regions and the amygdala (Stuhrmann et al., 2011). By contrast, high-level biases emerge in later cognitive control stages of processing involving the prefrontal cortex, and are thought to impact how emotional stimuli are interpreted and responded to (Fossati, 2008). Together, these biases reinforce negative schemata, perpetuating the symptoms of depression and anxiety (Bishop, 2007; Harmer et al., 2009; Roiser et al., 2012). Within this framework, selective serotonin reuptake inhibitors (SSRIs) are proposed to initially modulate low-level affective processing biases before changes in higher-level decisional biases and subsequent changes in mood occur (Godlewska and Harmer, 2021; Harmer et al., 2009; Roiser et al., 2012). Distinguishing these components is therefore critical for understanding how affective processing is modulated in mood disorders and targeted by treatments such as SSRIs.

One well-documented manifestation of affective biases is the tendency for individuals with depression and anxiety to categorise ambiguous facial expressions as more negative (Demenescu et al., 2010; Krause et al., 2021). Such biases in the interpretation of facial expressions of emotion have been shown to predict vulnerability to mood disorders, and to change early in antidepressant treatment (Godlewska et al., 2018; Tranter et al., 2009). Biases in the labelling of facial expressions can also be induced on shorter timescales with adaptation paradigms. In these paradigms, prior exposure to an “adaptor” stimulus biases the perception of subsequent stimuli away from the attributes of the adaptor, referred to as an aftereffect (Georgeson, 2004). For example, a bias towards reporting that ambiguous expressions appear sad can be induced after prolonged viewing of happy expressions (Rutherford et al., 2008). Thus, adaptation serves as a normative model for how the statistics of the world shape perception. Such effects generalise across changes in image size and orientation (Rhodes et al., 2004; Zhao and Chubb, 2001), ruling out low-level retinotopic processes. Originally dubbed “the psychologists microelectrode” (Frisby, 1979), adaptation aftereffects have been used as a tool in vision science to understand plasticity in neural representations for decades (Clifford et al., 2007). There is evidence to suggest that serotonin may facilitate the neural plasticity required for adaptation-induced perceptual biases to occur. Direct manipulation of serotonin in animals shows that fluoxetine alters the perceptual biases induced by adaptation (Bachatene et al., 2013), and 3,4-methylenedioxymethamphetamine models in humans also suggest that serotonergic functioning plays a key role in adaptation aftereffects (Dickson et al., 2009). However, no studies to date have capitalised on adaptation as a tool to understand the origin of emotional biases reported in affective disorders.

Any perceptual decision, however, is the result of processes involving the perceptual encoding of the stimulus and subsequent interpretation or decision-making processes. Subjective confidence ratings offer a novel method for disentangling these processes. Metacognitive research shows that people can accurately assess their own perceptual discrimination ability (Fleming and Lau, 2014), reporting high subjective confidence when the sensory evidence is unambiguous, while low confidence is associated with ambiguous sensory inputs. Across a series of experiments investigating adaptation aftereffects on motion perception, Gallagher et al. (2019) convincingly demonstrated that high uncertainty (i.e. low confidence) tracks the perceptual discrimination boundary (the Point of Subjective Equality (PSE)) following adaptation, but only in situations where the adaptation induces *perceptual* biases. Conversely, a shift in discrimination boundaries *without* any changes to uncertainty reflects biases in decisional processes. Thus, when confidence tracks or correlates with the biases induced by adaptation, those biases have a perceptual origin. This has been established across a series of elegant studies and replications, investigating biases in the perception of numerosity (Moscoso et al., 2020), motion (Gallagher et al., 2021), duration (Bruno et al., 2023) and face configuration (Gao et al., 2025). Here, we extend this approach to the domain of affective bias for the first time, asking whether negative affect influences the perceptual encoding of emotional expressions themselves, or only later decision-making processes. This represents a critical step towards linking basic visual adaptation mechanisms with cognitive-neuropsychological models of affective disorders.

Despite the key role of affective biases in neuropsychiatric conditions, it remains unclear whether biases in emotion inference from facial expressions observed in these conditions arise primarily from low-level perceptual processes or high-level decisional strategies. Distinguishing these contributions will provide further insight into neuropsychological models of mood disorders, which is important given their implications for understanding treatment mechanisms such as those targeted by SSRIs. In this study, we used an adaptation paradigm to induce both affective and non-affective biases and employed subjective confidence ratings to disentangle perceptual from decisional contributions. We also examined whether negative affect (a construct derived from shared features of depression and anxiety symptoms) modulated the relative contributions of these processes. Negative affect was derived using a latent variable approach, which is becoming more widely applied in characterising neuropsychiatric symptoms (Wise et al., 2023), with evidence to suggest that specific symptom scales to measure depression and anxiety may instead measure a more general “negative affect” (Andrade et al., 2001; Knowles and Olatunji, 2020). Building on extensive work showing adaptation effects in face perception, we hypothesise that adaptation will induce measurable biases in both emotion and identity judgements. If these biases reflect changes in perceptual encoding rather than decisional strategies, we expect corresponding shifts in confidence. Furthermore, if the mechanisms underlying these biases are shaped by individual differences in negative affect, we predict that the relationship between decision aftereffects and confidence will vary as a function of negative affect. Pinpointing where negative affect exerts its influence, for example, at the perceptual versus decisional level and in emotion versus identity judgements, will help clarify both the mechanistic origins and the domain specificity of these effects.

## Methods

### Participants

Eighty participants (47 females; age range: 18–45; mean age = 25.73 years, standard deviation (SD) = 6.40) were recruited via the Cambridge University participant recruitment system (https://www.sona-systems.com/). Three participants reported a diagnosis of major depressive disorder (MDD), and eight reported a familial diagnosis of MDD. Participants self-reported not being on any psychotropic medication at the time of testing.

Participants completed the State Trait Anxiety Inventory (STAI; Spielberger, 1983), which assesses both state (STAI-state) and trait (STAI-trait) anxiety, and the Beck Depression Inventory II (BDI-II; Beck et al.,1996). The distribution of scores was within the normal range for each measure (BDI: mean = 10.34, SD = 8.59; STAI-state: mean = 34.56, SD = 8.91; STAI-trait: mean = 42.60, SD = 10.98).

### Adaptation tasks

Participants completed identity and expression discrimination tasks, with two baseline and adaptation phases in each. The tasks were run in-person using Gorilla (www.gorilla.sc), using a 24.1 inch ASUS LCD monitor. Participants sat at a distance of 57 cm from the monitor with their head fixed in a chin rest.

**Figure 1.**
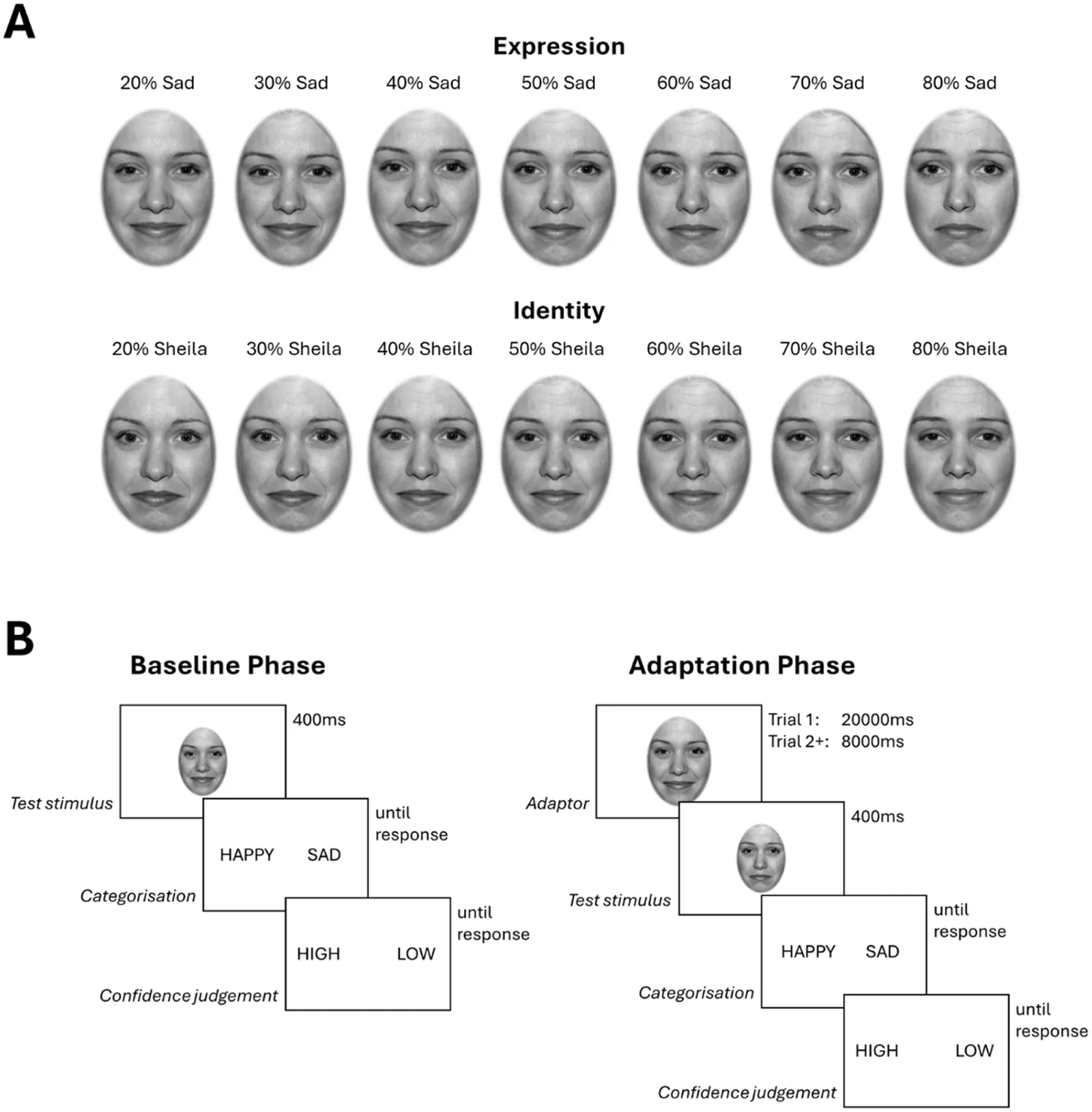
(a) Morphed expression and identity stimuli. Stimuli were orthogonalised across the tasks, such that the midpoint of the two identities was used in the expression discrimination task, and the midpoint of the two expressions was used in the identity discrimination task. (b) Trial sequences during baseline and adaptation phases. Figure shows examples from the emotion discrimination task.

Morphed facial stimuli were created by taking two female identities (“Bonny” and “Sheila”), each displaying happy and sad expressions, from the NimStim set of facial expressions (Tottenham et al., 2009). First, identity-independent expressions and expression-independent identities were created, by finding the average morph between the stimuli within expression and identity categories. For example, an identity-independent happy expression was created by taking the midpoint of the happy-Bonny and happy-Sheila morphs (Supplemental Figure 1). These faces were used as the “adaptor stimuli,” and morphs between the identity and expression categories were used to create “test stimuli.” For example, expression morphs (happy-to-sad) were created by taking seven morphs (at 20%, 30%, 40%, 50%, 60%, 70% and 80%) between the identity-independent happy and identity-independent sad expressions (Figure 1(a)). This ensured that the midpoint of the expression and identity continua were identical, so that the expression did not vary across the identity continuum and vice versa. All adaptor and test stimuli were greyscaled, luminance-matched and placed in an oval mask obscuring the features outside of the face.

In the baseline phases, participants were presented with the test stimuli (from either the identity or emotion dimensions) eight times each, along with the two adaptor faces four times each (a total of 64 stimuli), for 400 ms each, in a random order. After the presentation of each stimulus, participants made a binary judgement of expression (happy vs. sad) or identity (Bonny vs. Sheila), followed by a binary judgement of their confidence in their response (high vs. low).

In the adaptation phases, participants again provided binary judgements (of expression or identity, and confidence) following the presentation of each test stimulus; however, each test stimulus was preceded by an adaptor stimulus. The first adaptor stimulus within this phase was presented for 20,000 ms, and subsequent adaptors were presented for 8000 ms to “top-up” the effects of adaptation. Trial sequences are presented in Figure 1(b). The seven test stimuli were each presented eight times in a random order, for 400 ms each. To minimise retinotopic contributions to any adaptation effects, the test stimuli subtended 5° × 7° of visual angle, while the adaptor stimuli subtended 8° × 9°.

The task was completed in four blocks (one per adapting stimulus), where each block comprised a baseline phase followed by an adaptation phase. For each adapting stimulus, participants judged both the expression and identity of the test stimuli. Block order was counterbalanced across participants.

### Analysis

For each participant and condition, logistic functions were fit to describe the proportion of “sad” responses (in the expression discrimination task) and “Sheila” responses (in the identity discrimination task) across the stimulus morphs, where parameters µ_logistic_ and σ_logistic_ describe the PSE and slope, respectively. The PSE is the point along the x-axis where the value of the logistic function = 0.5. That is, it is the stimulus along the continuum of morphed faces that an observer is equally likely to categorise as belonging to either category. The proportions of “low” confidence judgements for each stimulus morph were fit with Gaussian functions, where parameters µ_Gaussian_ and σ_Gaussian_ describe the peak uncertainty and the “uncertainty spread,” respectively. Functions were fit using the *lmfit* package in Python (https://lmfit.github.io/lmfit-py/). If either µ parameter fell outside the bounds [0, 100] the datapoint was excluded. As participants completed a baseline phase before each of the two adaptation phases for each discrimination task, the data for the two baseline phases were concatenated before fitting the functions. Effects of adaptation were calculated as the difference between the function parameters in the adaptation and baseline conditions.

A latent negative affect dimension was derived from the STAI-trait and BDI scores with principal component analysis after scaling and standardising each dimension (to a mean of 0 and SD of 1), using the Principal Components Analysis (PCA) function in the sklearn package in Python (https://scikit-learn.org/). This approach follows evidence that depressive and anxious symptoms largely reflect a common negative affect dimension (Andrade et al., 2001; Knowles and Olatunji, 2020; Wise et al., 2023). We did not administer a direct negative affect scale (e.g. the Positive and Negative Affect Schedule), as our goal was to quantify trait-level affective vulnerability rather than self-reported mood states. We note that we did not include state anxiety to derive the negative affect dimension, as we were primarily interested in trait effects.

To assess whether the induced biases were perceptual or decisional, correlations were run between the effects of adaptation on the parameters describing PSEs (µ_logistic_) and peak uncertainties (µ_gaussian_). We next tested whether negative affect interacts with this relationship by using ordinary least squares regression to predict changes in µ_logistic_ using changes in µ_gaussian_, including a term to model the interaction between change in µ_gaussian_ with negative affect, while controlling for µ_logistic_ at baseline to account for any effects of baseline PSE on adaptation magnitude.

## Results

### Identification of negative affect

**Figure 2.**
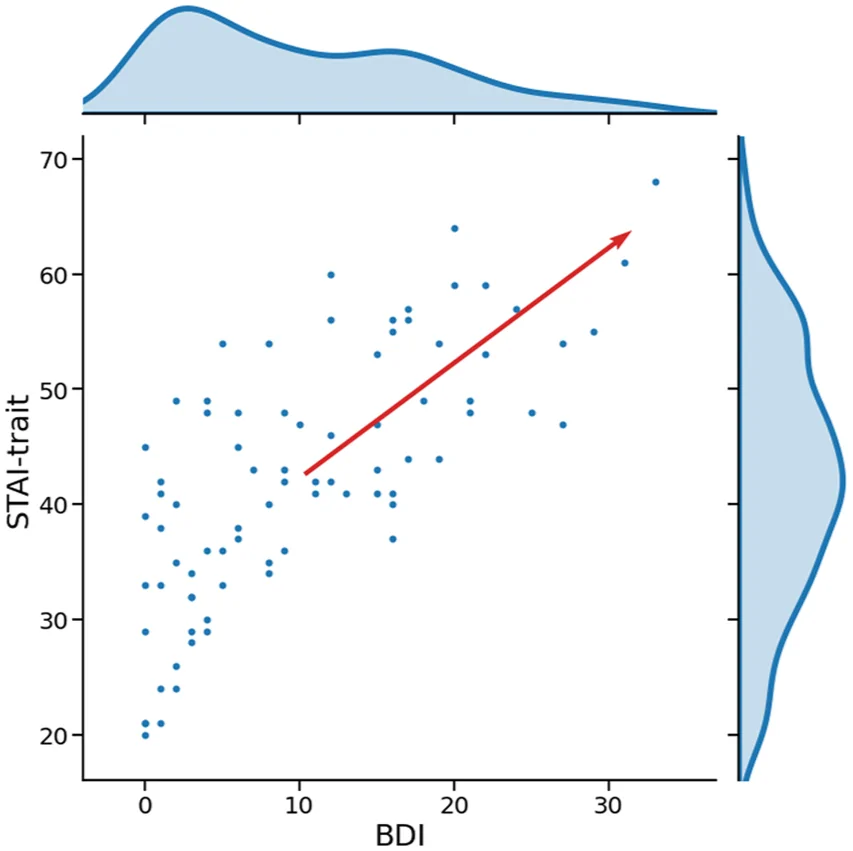
Scatterplot and densities of BDI and STAI-trait scores. The first principal component is indicated by the red arrow. The scores along this dimension are taken as a measure for “negative affect.” BDI: Beck Depression Inventory; STAI: State Trait Anxiety Inventory.

Trait anxiety and BDI were strongly positively correlated (*r*(79) = 0.736, *p* < 0.001), suggesting shared symptoms. Both measures were scaled to a mean of 0 and SD of 1, then PCA was used to identify a single latent component. This component explained 86.6% of the total variance, with an Eigenvalue of 1.758, suggesting this dimension fits the two symptom dimensions well. Unscaled BDI and STAI-trait scores are presented in Figure 2, with the first principal component. Exploratory t-tests revealed there were no sex differences in BDI, STAI-trait or the derived negative affect dimension (all *p*’s > 0.1).

### Effects of adaptation

Both sets of functions fit the data well. Table S1 shows a summary of the fits of the logistic and Gaussian functions across the different conditions.

We observed significant adaptation-induced biases on the PSEs of the psychometric functions across both identities and expressions. The threshold to label sad faces was significantly smaller than baseline after adapting to happy faces (*t*(77) = 5.705, *p* < 0.001, *d* = 0.646), and larger after adapting to sad faces (*t*(76) = 19.178, *p* < 0.001, *d* = 2.186). In other words, adapting to happy faces produced a bias towards sad responses, whereas adapting to sad faces produced a bias towards happy responses. Similarly, the threshold to label Sheila was significantly smaller than baseline after adapting to Bonny (*t*(70) = 11.275, *p* < 0.001, *d* = 1.338), and larger after adapting to Sheila (*t*(67) = 8.129, *p* < 0.001, *d* = 0.986). Again, adapting to Sheila produced a bias towards more Bonny responses, and vice versa. These biases are consistent with the expected repulsive aftereffects that result from adaptation.

**Figure 3.**
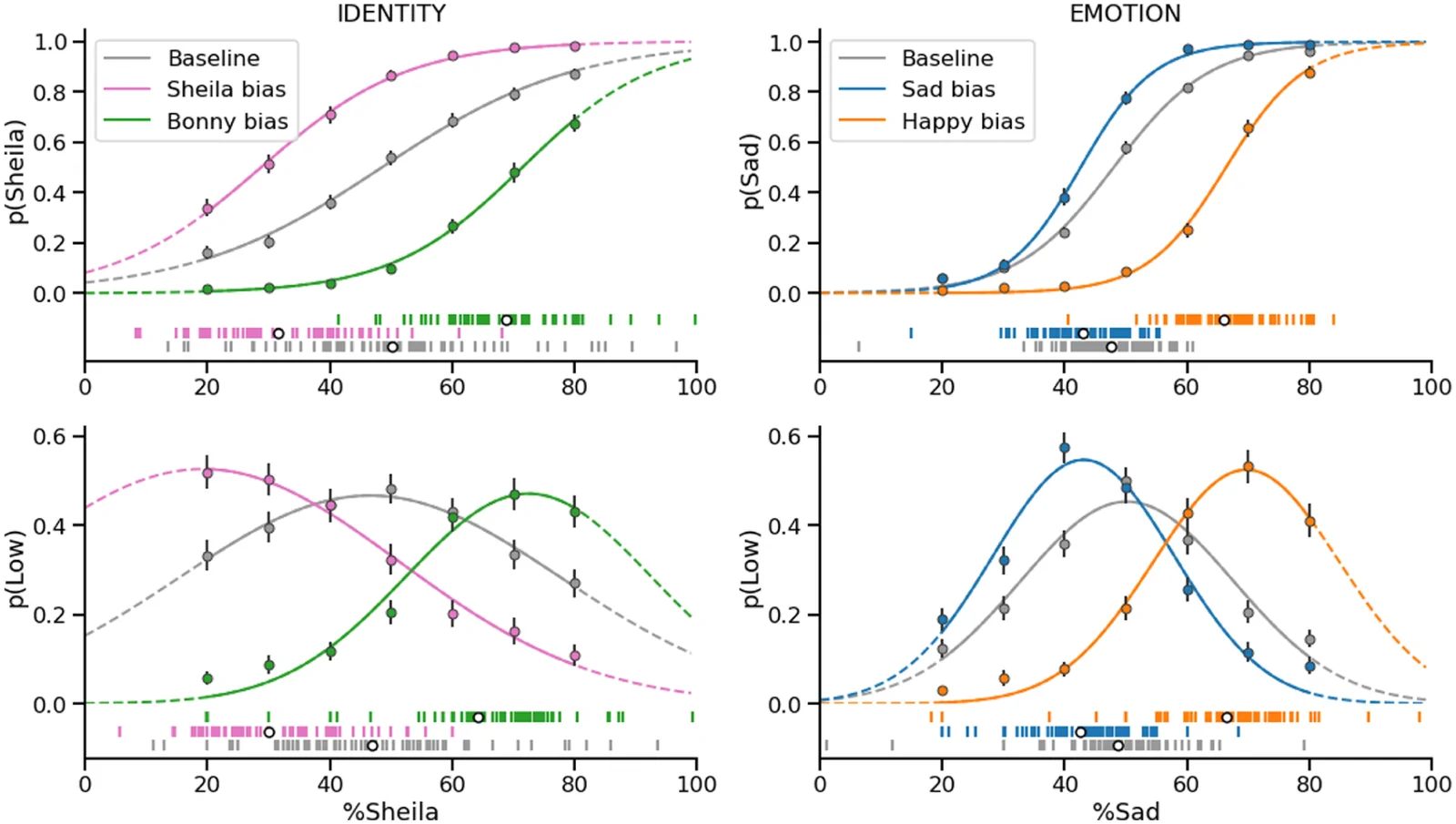
Logistic (upper) and Gaussian (lower) functions, fit to the average responses in each of the different identity (left) and emotion (right) discrimination conditions. X-axes show the morph level of the face (%Sheila for the identity task and %Sad for the emotion task), and Y-axes show the proportion of responses to each morph level (p(Sheila) for identity and p(Sad) for emotion). The top left shows that following adaptation to the Bonny identity, perception of the test stimuli shifts away from Bonny (a bias towards Sheila). Similarly, perception is biased towards Bonny (away from Sheila) following adaptation to Sheila. Bottom left shows that confidence judgements following adaptation tend to shift in line with the aftereffect. This suggests a change in perception, rather than decisional processes. The top right shows a similar pattern of aftereffects following emotion adaptation, with perception shifting away from happy expressions (a bias towards sad) following adaptation to happy faces, and the opposite bias following adaptation to sad faces (i.e. a shift in perception towards happy). Again, confidence appears to track the adaptation aftereffects. Error bars on data points show standard errors. µ parameters (centre points) of each function for each participant are shown below each function, along with the mean of these points across participants.

We also observed similar adaptation-induced biases on peak uncertainty across both identities and expressions. The position of the peak uncertainty (along the x-axis) was significantly smaller than baseline after adapting to happy faces (*t*(74) = 5.337, *p* < 0.001, *d* = 0.616), and significantly larger after adapting to sad faces (*t*(71) = 14.209, *p* < 0.001, *d* = 1.675). Similarly, the position of the peak uncertainty was significantly smaller than baseline after adapting to the Bonny (*t*(65) = 8.630, *p* < 0.001, *d* = 1.062), and larger than baseline after adapting to Sheila (*t*(65) = 6.995, *p* < 0.001, *d* = 0.861). Functions fit to averaged responses are presented in Figure 3.

Exploratory t-tests revealed there were no sex differences in the effects of adaptation on any PSEs or peak uncertainty parameters (all *p*’s > 0.1).

For the PSE and peak uncertainty, we calculated the strength of the aftereffect per participant in the happy, sad, Bonny and Sheila adaptation conditions as the parameter in the adaptation block minus the parameter in the averaged baseline blocks (using averaged emotion baseline to calculate the happy and sad aftereffects, and the averaged identity baseline to calculate the Bonny and Sheila aftereffects).

**Figure 4.**
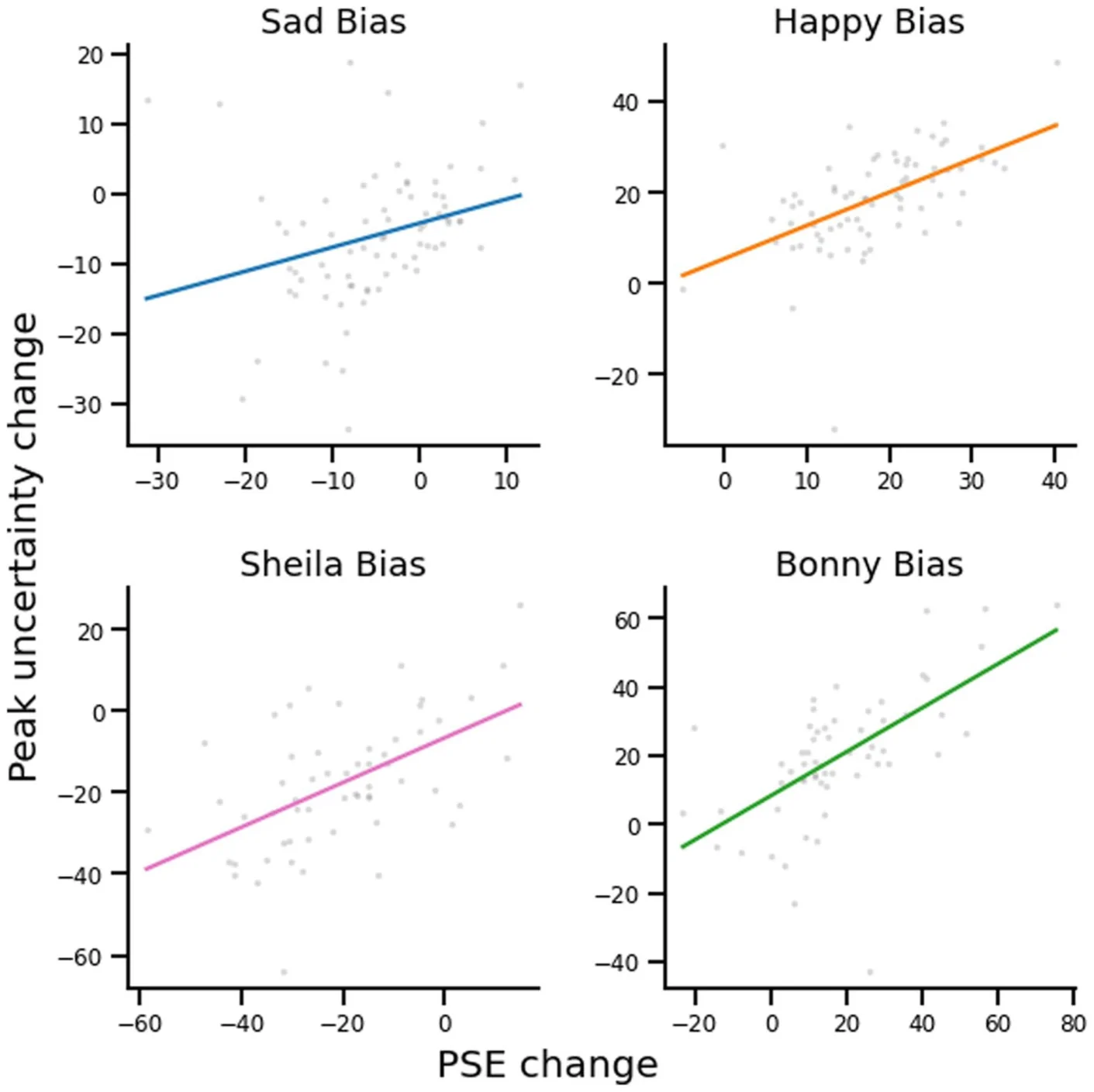
Scatterplots showing the correlations between PSE change and peak-uncertainty change following adaptation, for the two identity and two affective conditions. Each plot shows the bias towards a response category after adapting to the opposite stimulus (e.g. “Sad bias” shows the changes in PSE and peak uncertainty in the happy-adaptation condition). PSE: point of subjective equality.

We found that adaptation effects on PSE and peak uncertainty were positively correlated in all adaptation conditions (happy adaptation: *r*(74) = 0.284, *p* = 0.014; sad adaptation: *r*(71) = 0.543, *p* < 0.001; Bonny adaptation: *r*(56) = 0.535, *p* < 0.001; Sheila adaptation: *r*(58) = 0.613, *p* < 0.001), suggesting that the identity and expression biases induced by adaptation affect perceptual rather than decisional processes across the sample (Figure 4).

### Interaction between negative affect and induced biases

Negative affect was not correlated with the simple change in any perceptual discrimination threshold following adaptation (happy adaptation: *r*(77) = 0.212, *p* = 0.062; sad adaptation: *r*(76) = −0.172, *p* = 0.134; Bonny adaptation: *r*(70) = −0.114, *p* = 0.342; Sheila adaptation: *r*(67) = −0.032, *p* = 0.796), suggesting no relationship between negative affect and the magnitude of any induced affective or non-affective biases. Similarly, negative affect was not correlated with the change in peak uncertainty following adaptation (happy adaptation: *r*(74) = −0.162, *p* = 0.164; sad adaptation: *r*(71) = −0.034, *p* = 0.775; Bonny adaptation: *r*(65) = −0.039, *p* = 0.757; Sheila adaptation: *r*(65) = −0.032, *p* = 0.799).

**Figure 5.**
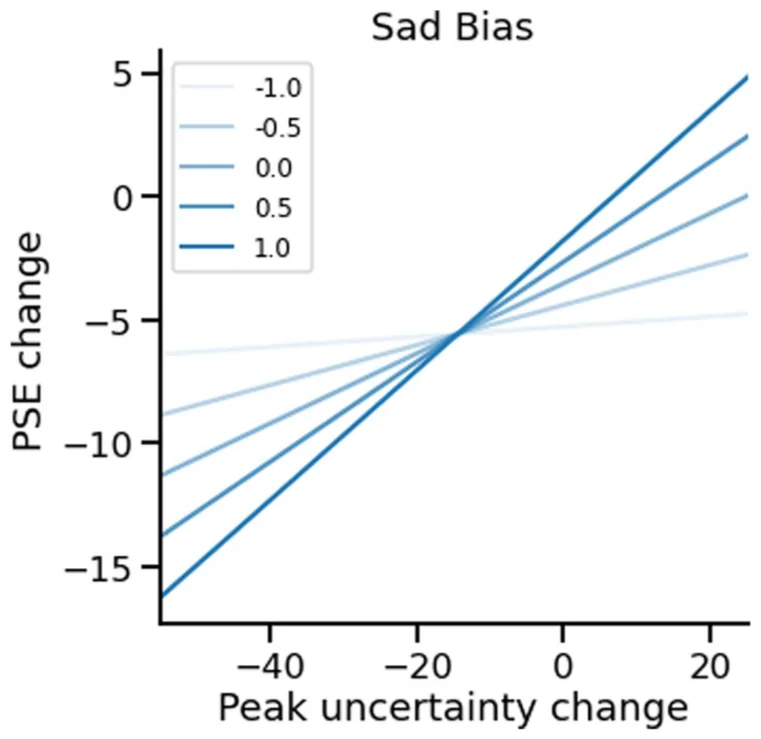
Plot showing the interaction in the regression model for induced sad biases. Separate lines show the predicted change in PSE (y-axis) given changes in peak uncertainty (x-axis), for different values of negative affect, showing that as negative affect increases (pale blue increasing to darker blue), peak uncertainty tracks PSE change more closely – shifting the balance from decisional towards more perceptual biases. PSE: point of subjective equality.

We next tested whether negative affect interacts with the *relationship between* PSE change and peak uncertainty change, using ordinary least squares regression. For happy adaptation (i.e. a bias towards more sad interpretations of faces), we found a significant positive interaction between negative affect and the relationship between the bias strength on PSE and peak uncertainty (β = 0.122, *t* = 2.292, *p* = 0.025). This suggests that, for those high in negative affect, the induced negativity bias following happy adaptation affects perceptual processes more than decisional (see Figure 5). No interaction terms in the other models were significant (sad adaptation: β = −0.006, *t* = −0.098, *p* = 0.922; Bonny adaptation: β = 0.107, *t* = 1.546, *p* = 0.128; Shiela adaptation: β = −0.063, *t* = −0.961, *p* = 0.341), suggesting that negative affect interacts with perceptual processes involved in negative affective biases specifically. Full outputs from each of the regression models are reported in the Supplemental Materials.

## Discussion

In this study, we aimed to examine whether affective biases, similar to those observed in neuropsychiatric conditions, have perceptual or decisional origins. Specifically, we used an adaptation paradigm to induce both affective (emotion) and non-affective (identity) biases in the judgement of faces, and used subjective confidence ratings to disentangle the role of perceptual processing from decision-making strategies (Gallagher et al., 2019). We observed repulsive aftereffects, such that adaptation to an expression or identity induced a bias in the reported perception of subsequent stimuli that was in the opposite direction to the adaptor stimulus. These biases were measured in both changes in the PSE and the peak uncertainty relative to baseline, and these changes were correlated, consistent with perceptual rather than decisional processes. Importantly, we found that negative affect modulated this relationship for biases towards labelling expressions as sad, such that the relationship between adaptation effects on PSE and peak uncertainty was higher for those high in negative affect. This effect was not observed for induced positive affective biases or non-affective biases, suggesting specificity to negative affective biases. These findings provide novel evidence that affective biases – particularly towards negative interpretations of facial emotion – may emerge from changes in early perceptual encoding, rather than higher-level decision strategies. This distinction has important implications for theories of affective disorders and their treatment.

These results align with cognitive neuropsychological models of mood and anxiety disorders that posit low-level or bottom-up negative affective biases play a key role in the development and maintenance of negative schemata (Bishop, 2007; Roiser et al., 2012). According to these models, SSRIs initially target low-level perceptual biases by reducing amygdala hyperactivity, causing gradual positive changes in perception of socio-emotional cues. This eventually leads to a downstream reduction of higher-level decisional biases and eventual improvement in mood symptoms. As we found that the negativity biases (induced by adaptation) were caused by changes to perceptual processes rather than decisional, we would expect the strength of this bias to reduce following acute administration of an SSRI. Indeed, this would align with previous findings that acute SSRI administration reduces amygdala responses to emotional stimuli and biases attention and perception away from threat (Godlewska et al., 2012; Harmer et al., 2006). It is also possible that SSRI effects on adaptation-induced emotional biases might arise via the visual system, given prior work suggesting that serotonin directly affects the gain control of neurons in early visual cortex – with subsequent impacts on behavioural adaptation aftereffects (Azimi et al., 2020; Seillier et al., 2017). More speculatively, our findings raise the possibility that adaptation-induced perceptual biases might be used as a behavioural marker to predict antidepressant response, complementing neural or clinical predictors (Godlewska et al., 2018). However, this hypothesis requires direct testing in follow-up psychopharmacological studies.

Neuropsychological models of the brain systems supporting emotion perception suggest that the amygdala (and other regions in the limbic system) are involved with salience detection, whereas prefrontal regions are involved with emotion regulation (Phillips et al., 2003a). These models suggest that mood disorders are associated with relative hyperactivity in the salience network and relative underactivity in the regulation network (Phillips et al., 2003b) – a suggestion that is supported by a meta-analysis of neuroimaging studies with MDD patients (Hamilton et al., 2012). An initial effect of SSRIs is the reduction in low-level perceptual biases, which is argued to be the result of a reduction of amygdala hyperactivity (Godlewska et al., 2012; Harmer et al., 2006; Murphy, 2010; Pringle et al., 2011). According to these models, the induced negative affective biases following adaptation in our results would therefore be associated with a relative hyperactivity in the amygdala, rather than an underactivity in the prefrontal cortex. One study using functional magnetic resonance imaging with an adaptation paradigm provides evidence for this, showing that the same expression image caused greater activation in the amygdala and insula following adaptation to happy faces (therefore inducing a negative affective bias) than following adaptation to sad faces, but found no effects of adaptation on prefrontal activity (Su et al., 2024). An interesting question for future research would be to examine whether the adaptation-induced differences in amygdala activation are modulated by neuropsychiatric traits.

We found repulsive aftereffects consistent with a wealth of literature using adaptation paradigms in the context of identity and emotion perception (Campbell and Burke, 2009; Ellamil et al., 2008; Fox and Barton, 2007; Furl et al., 2007; Leopold et al., 2001; Moradi et al., 2005; Rhodes et al., 2015; Rutherford et al., 2008; Skinner and Benton, 2012; Song et al., 2015; Webster et al., 2004; Zhao and Chubb, 2001). However, these studies have not considered whether the observed aftereffects are caused by perceptual or decision-making biases. Our results add to this literature by showing that both identity and expression aftereffects are primarily caused by changes in perceptual encoding, rather than shifts in decisional criteria. We followed the approach introduced by Gallagher et al. (2019), who demonstrated that subjective confidence, when measured alongside categorical judgements, can serve as a diagnostic tool to distinguish between perceptual and decisional sources of aftereffects. Specifically, perceptual aftereffects are expected to alter both the PSE and the point of peak uncertainty, while purely decisional biases shift the PSE without affecting confidence. Using this logic, we found that adaptation shifted both PSEs and uncertainty in tandem, supporting a perceptual origin. This approach has since been applied across a range of perceptual domains, including numerical cognition (Moscoso et al., 2020), motion perception (Bruno et al., 2023; Gallagher et al., 2021) and face distortions (Gao et al., 2025) – all converging on the conclusion that many high-level aftereffects previously assumed to reflect post-perceptual biases may, in fact, involve changes in perceptual representations.

Interestingly, we found that negative affect did not influence the overall magnitude of the aftereffects, but instead modulated their underlying locus – shifting the balance towards perceptual rather than decisional contributions. This suggests that individuals higher in negative affect may not experience *more* bias in their interpretations of emotional expressions, but rather experience a different *quality* of bias: one that reflects changes in how stimuli are encoded, rather than how they are interpreted or classified. Given that perceptual adaptation can arise at multiple stages – ranging from low-level changes in contrast gain and tuning (Solomon and Kohn, 2014), through mid-level configural encoding of faces (Rhodes and Leopold, 2011), to shifts in categorical boundaries or representational geometry (Clifford et al., 2007; Webster et al., 2004) – our findings raise the possibility that affect selectively modulates one or more of these perceptual layers. This interpretation is consistent with evidence that affective states can sharpen or weight specific features of visual input (Aday and Carlson, 2019), and with models suggesting that antidepressant treatment initially targets these early biases (Harmer et al., 2009). The fact that this effect was specific to negative, and not positive or non-affective biases, suggests that the visual system may be particularly tuned to encode negatively valenced social cues with higher fidelity or salience under conditions of elevated negative affect. This is consistent with theoretical accounts of affective bias outside the social domain (Browning et al., 2010; Pulcu and Browning, 2019). This may help explain why individuals with anxiety and depression often report a sense of certainty when interpreting ambiguous expressions as threatening or sad.

More broadly, our findings align with theoretical accounts in which emotional states influence perception by shaping prior expectations or the weight placed on particular sensory features. Rather than simply biasing interpretive strategies, negative affect may recalibrate the perceptual system itself; for example, by increasing sensitivity to features associated with sadness or threat. This kind of modulation could amplify the perceived salience of affectively congruent signals, without increasing overall bias magnitude. From a clinical perspective, this suggests that behavioural measures capable of dissociating perceptual from decisional biases may provide a sensitive window into early, treatment-relevant features of affective processing. Future work using neuroimaging or pharmacological interventions could test whether this perceptual weighting is reflected in differential activity across emotion-responsive visual and limbic regions, and whether it changes with mood state or clinical response.

While our findings provide evidence for perceptual origins of affective bias, they were obtained in a predominantly healthy sample, with only three participants reporting a diagnosis of MDD. While this allowed us to examine dimensional effects of negative affect, mean BDI and STAI scores fell within the nonclinical range, restricting variability and potentially attenuating associations with negative affect. As such, the findings are best interpreted as reflecting individual differences in affective bias within the healthy spectrum rather than direct analogues of clinical processes. Future work should test whether similar perceptual-decisional dissociations emerge in clinical populations with diagnosed mood or anxiety disorders, and on the basis of the current results, we would speculate that people with diagnosed affective disorders would show stronger perceptual biases towards sadness. Studies in larger clinical samples could also examine how medication status influences these effects, or use controlled pharmacological manipulations to causally test the role of serotonergic modulation in potentially reversing these affective biases or shifting their locus of origin.

One methodological consideration is that participants were unfamiliar with the two target identities used in our study, whereas the emotional expressions (happy and sad) were highly familiar and well-practised categories. This asymmetry may have influenced both confidence ratings (where participants gave more “low” confidence responses in the identity tasks) and overall task performance, as reflected in shallower psychometric slopes for identity compared to expression discrimination. Importantly, our key findings are based on shifts in the PSE, rather than overall discrimination sensitivity, making it unlikely that these baseline task differences confounded the observed aftereffects. Additionally, participants were presented with images of “Bonny” and “Sheila” at the beginning of the study to familiarise them with the two identities before beginning the study. Nonetheless, future work could more directly test this assumption by equating familiarity across tasks, for example, through an extended pre-adaptation familiarisation protocol designed to stabilise identity representations until behavioural discrimination performance matches that observed for expression.

While visual adaptation provides a powerful tool for probing perceptual plasticity, the ecological validity of adaptation effects warrants consideration. In everyday life, emotional biases are not typically induced by prolonged exposure to a single expression, but rather emerge through accumulated experience and contextual learning. Nevertheless, adaptation serves as a normative model of how recent sensory history shapes perceptual encoding (Clifford et al., 2007). Repeated or systematic recalibration of affective perception (analogous to adaptation) could conceivably form the basis for perceptual retraining interventions, though this remains speculative. Our findings therefore clarify how affective experience influences perceptual encoding, providing a mechanistic foundation that future work can build on to examine these processes over longer timescales and therapeutic settings.

This study provides compelling evidence that affective biases in emotion perception originate from early-stage perceptual encoding, rather than from higher-order decisional strategies. Crucially, we show that adaptation-induced negativity biases reflect distortions in how emotional expressions are *perceived*, not merely how they are interpreted or reported – and that this effect is especially pronounced in individuals with elevated negative affect. These findings challenge the notion that affective biases are purely cognitive or interpretive, and instead position perceptual mechanisms as a central, modifiable substrate of emotion processing.

## Supplemental Material

sj-docx-1-jop-10.1177_02698811251409143 – Supplemental material for Negative affect interacts with perceptual affective biases Supplemental material, sj-docx-1-jop-10.1177_02698811251409143 for Negative affect interacts with perceptual affective biases by Thomas Murray and Rebecca P. Lawson in Journal of Psychopharmacology
