# Supplementary material for: Negative affect interacts with perceptual affective biases
Source: J Psychopharmacol. 2026 Feb 1;40(9):1405–14. doi: 10.1177/02698811251409143 (PMC13490696; doi:10.1177/02698811251409143)
Supplement: Supplementary file 1 [file sj-docx-1-jop-10.1177_02698811251409143.docx]

Negative affect interacts with perceptual affective biases - Supplementary Information

*Supplementary Figure 1: Identity-independent expression stimuli (in blue frames) and expression-independent identity stimuli (in orange frames) were created from the original stimuli, and were used to create the test stimuli in the task.*

# Model fits

Table S1 shows a summary of the fits of the logistic and gaussian functions across the different conditions.

*Table S1: Summary of model fits to the responses in the different conditions.*

| **Condition** | **Function** | **N fit (%)** | **Mean R^2^** |
| --- | --- | --- | --- |
| Emotion baseline | Logistic | 80 (100%) | 0.959 |
| Happy-adapt | Logistic | 78 (97%) | 0.978 |
| Sad-adapt | Logistic | 77 (96.2%) | 0.948 |
| Identity baseline | Logistic | 77 (96.2%) | 0.893 |
| Bonny-adapt | Logistic | 71 (88.8%) | 0.922 |
| Sheila-adapt | Logistic | 70 (87.5%) | 0.929 |
| Emotion baseline | Gaussian | 77 (96.2%) | 0.820 |
| Happy-adapt | Gaussian | 77 (96.2%) | 0.887 |
| Sad-adapt | Gaussian | 74 (92.5%) | 0.899 |
| Identity baseline | Gaussian | 74 (92.5%) | 0.721 |
| Bonny-adapt | Gaussian | 72 (90.0%) | 0.815 |
| Sheila-adapt | Gaussian | 70 (87.5%) | 0.862 |

# Interactions between negative affect and induced biases

Below are the full outputs for the regressions examining the interaction between negative affect and the relationship between adaptation effects on peak uncertainty and PSE. For all regressions, the adaptation induced change in PSE (Δ PSE) was predicted using the adaptation induced change in peak uncertainty (Δ Peak uncertainty), negative affect, the interaction between the change in peak uncertainty and negative affect, and the baseline PSE for that task (i.e. the baseline emotion PSE or baseline identity PSE), using the formula:

$$\Delta PSE=\beta_{0}+\beta_{1}\Delta Peak Uncertainty+\beta_{2}Negative affect+\beta_{3}\left( \Delta Peak Uncertainty*Negative affect \right)+\beta_{4}Baseline PSE+\epsilon$$

Variance Inflation Factors (VIF) were calculated to assess multicollinearity among the predictors. We note that all VIFs fall below the recommended threshold of 5.

## Happy aftereffects (Sad bias)

*Table S2: Regression on happy aftereffects; model fit*

| N observations | 75 |
| --- | --- |
| DF Residuals | 70 |
| DF Model | 4 |
| R^2^ | 0.421 |
| F | 12.73 |
| p | <.001 |
| VIF | 1.494 |

*Table S3: Regression on happy aftereffects; coefficients*

|  | **Coeff** | **Std Err** | **t** | **p** | **[.025** | **.975]** |
| --- | --- | --- | --- | --- | --- | --- |
| Intercept | 29.195 | 6.223 | 4.692 | <.001 | 16.784 | 41.605 |
| Δ Peak uncertainty | 0.142 | 0.081 | 1.750 | .084 | -0.020 | 0.304 |
| Negative affect | 1.746 | 0.662 | 2.658 | .010 | 0.436 | 3.066 |
| Δ Peak uncertainty * Negative affect | 0.122 | 0.053 | 2.292 | .025 | 0.016 | 0.227 |
| Emotion baseline PSE | -0.688 | 0.131 | -5.250 | <.001 | -0.950 | -0.427 |

## Sad aftereffects (Happy bias)

*Table S4: Regression on sad aftereffects; model fit*

| N observations | 72 |
| --- | --- |
| DF Residuals | 67 |
| DF Model | 4 |
| R^2^ | 0.483 |
| F | 15.62 |
| p | <.001 |
| VIF | 3.390 |

*Table S5: Regression on sad aftereffects; coefficients*

|  | **Coeff** | **Std Err** | **t** | **p** | **[.025** | **.975]** |
| --- | --- | --- | --- | --- | --- | --- |
| Intercept | 39.224 | 6.404 | 6.125 | <.001 | 26.442 | 52.005 |
| Δ Peak uncertainty | 0.329 | 0.067 | 4.899 | <.001 | 0.195 | 0.462 |
| Negative affect | -1.356 | 1.213 | -1.118 | .268 | -3.777 | 1.065 |
| Δ Peak uncertainty * Negative affect | -0.006 | 0.058 | -0.098 | .922 | -0.120 | 0.109 |
| Emotion baseline PSE | -0.570 | 0.124 | -4.594 | <.001 | -0.818 | -0.322 |

## Bonny aftereffects (Sheila bias)

*Table S6: Regression on Bonny aftereffects; model fit*

| N observations | 57 |
| --- | --- |
| DF Residuals | 52 |
| DF Model | 4 |
| R^2^ | 0.562 |
| F | 16.67 |
| p | <.001 |
| VIF | 2.806 |

*Table S7: Regression on sad aftereffects; coefficients*

|  | **Coeff** | **Std Err** | **t** | **p** | **[.025** | **.975]** |
| --- | --- | --- | --- | --- | --- | --- |
| Intercept | 13.826 | 5.128 | 2.696 | .009 | 3.535 | 24.117 |
| Δ Peak uncertainty | 0.240 | 0.103 | 2.332 | .024 | 0.034 | 0.447 |
| Negative affect | 1.121 | 1.626 | 0.689 | .494 | -2.143 | 4.384 |
| Δ Peak uncertainty * Negative affect | 0.107 | 0.069 | 1.546 | .128 | -0.032 | 0.246 |
| Identity baseline PSE | -0.572 | 0.106 | -5.423 | <.001 | -0.784 | -0.360 |

## Sheila aftereffects (Bonny bias)

*Table S8: Regression on Bonny aftereffects; model fit*

| N observations | 59 |
| --- | --- |
| DF Residuals | 54 |
| DF Model | 4 |
| R^2^ | 0.711 |
| F | 33.26 |
| p | <.001 |
| VIF | 1.355 |

*Table S9: Regression on sad aftereffects; coefficients*

|  | **Coeff** | **Std Err** | **t** | **p** | **[.025** | **.975]** |
| --- | --- | --- | --- | --- | --- | --- |
| Intercept | 52.910 | 6.218 | 8.510 | <.001 | 40.444 | 65.376 |
| Δ Peak uncertainty | 0.259 | 0.082 | 3.168 | .003 | 0.095 | 0.423 |
| Negative affect | 1.739 | 1.655 | 1.051 | .298 | -1.580 | 5.058 |
| Δ Peak uncertainty * Negative affect | -0.063 | 0.065 | -0.961 | .341 | -0.194 | 0.068 |
| Identity baseline PSE | -0.791 | 0.100 | -7.880 | <.001 | -0.993 | -0.590 |
